# The lncRNA *TP73-AS1* is linked to aggressiveness in glioblastoma and promotes temozolomide resistance in glioblastoma cancer stem cells

**DOI:** 10.1038/s41419-019-1477-5

**Published:** 2019-03-13

**Authors:** Gal Mazor, Liron Levin, Daniel Picard, Ulvi Ahmadov, Helena Carén, Arndt Borkhardt, Guido Reifenberger, Gabriel Leprivier, Marc Remke, Barak Rotblat

**Affiliations:** 1 Department of Life Sciences, Ben-Gurion University of the Negev, Beer-Sheva, Israel; 2 Bioinformatics Core Facility, National Institute for Biotechnology in the Negev, Ben-Gurion University of the Negev, Beer-Sheva, Israel; 3 Department of Pediatric Neuro-Oncogenomics, German Cancer Research Center (DKFZ), Heidelberg, Germany; 4 Department of Pediatric Oncology, Hematology, and Clinical Immunology, Medical Faculty, University Hospital Düsseldorf, Düsseldorf, Germany; 5 Institute of Neuropathology, Medical Faculty, University Hospital Düsseldorf, Düsseldorf, Germany; 6 German Cancer Consortium (DKTK), partner site Essen/Düsseldorf, Germany; 7 Sahlgrenska Cancer Center, Department of Pathology, Institute of Biomedicine, Sahlgrenska Academy, University of Gothenburg, Gothenburg, Sweden

## Abstract

Glioblastoma multiform (GBM) is the most common brain tumor characterized by a dismal prognosis. GBM cancer stem cells (gCSC) or tumor-initiating cells are the cell population within the tumor-driving therapy resistance and recurrence. While temozolomide (TMZ), an alkylating agent, constitutes the first-line chemotherapeutic significantly improving survival in GBM patients, resistance against this compound commonly leads to GBM recurrence and treatment failure. Although the roles of protein-coding transcripts, proteins and microRNA in gCSC, and therapy resistance have been comprehensively investigated, very little is known about the role of long noncoding RNAs (lncRNAs) in this context. Using nonoverlapping, independent RNA sequencing and gene expression profiling datasets, we reveal that *TP73-AS1* constitutes a clinically relevant lncRNA in GBM. Specifically, we demonstrate significant overexpression of *TP73-AS1* in primary GBM samples, which is particularly increased in the gCSC. More importantly, we demonstrate that *TP73-AS1* comprises a prognostic biomarker in glioma and in GBM with high expression identifying patients with particularly poor prognosis. Using CRISPRi to downregulate our candidate lncRNA in gCSC, we demonstrate that *TP73-AS1* promotes TMZ resistance in gCSC and is linked to regulation of the expression of metabolism- related genes and ALDH1A1, a protein known to be expressed in cancer stem cell markers and protects gCSC from TMZ treatment. Taken together, our results reveal that high *TP73-AS1* predicts poor prognosis in primary GBM cohorts and that this lncRNA promotes tumor aggressiveness and TMZ resistance in gCSC.

## Introduction

Glioblastoma multiform (GBM) is the most common primary tumor of the central nervous system (CNS) with a dismal outcome and a 5-year overall survival rate of <10%^1^. Despite multimodal therapeutic strategies encompassing surgical resection, radiation, and temozolomide (TMZ)-based chemotherapy^2^, GBM constitutes a major clinical challenge. This is due to its tendency to the infiltrative growth pattern and therapy resistance, both resulting in high recurrence rates, and eventually, therapeutic failure. A major advancement in deciphering GBM biology was the identification of glioblastoma multiform cancer stem cells (gCSC)^3–5^. These cells were shown to drive self-renewal, invasive GBM growth, and therapy resistance^6,7^. Therefore, numerous studies have focused on characterizing and targeting gCSC^6,8^.

To improve cure rates for GBM patients, a better understanding of the genetic and transcriptional events promoting tumor cell growth, survival, and drug resistance is urgently required^9^. While significant progress has been made in delineating the functions of protein-coding genes and microRNA in GBM biology, the functions of long noncoding RNAs (lncRNAs) in this disease are beginning to be elucidated. In one such study, a clinically relevant lncRNA, namely *HIF1A-AS2*, was found to promote stemness and tumorigenicity in mesenchymal gCSC^10^. However, the role and relevance of the vast majority of lncRNAs in GBM are currently unknown.

LncRNAs are RNA transcripts longer than 200 base pairs (bp) that do not code for proteins^11^. They are transcribed from genes with functional, well-conserved promoters^12,13^ that are bound by various transcription factors and that exhibit histone methylation patterns similar to those found in protein-coding genes^14^. The human genome harbors ~21,000 protein-coding genes and more than 50,000 lncRNA genes^15^, which are very poorly characterized to date. Nevertheless, in the past few years, lncRNAs have been found to play important roles in all aspects of cell biology, including stemness, immunity, development, regulation of gene expression, regulation of protein synthesis, and in various diseases^16–19^.

Recent studies suggest that the lncRNA *TP73-AS1* may play a pivotal role in brain cancer biology. Specifically, DNA methylation of the *TP73-AS1* promoter was reported to confer epigenetic downregulation of its expression in oligodendroglial tumors compared with the normal brain^20^. As part of a lncRNA-based signature, the expression of *TP73-AS1* has been correlated with poor patient outcome in GBM^21^. Other studies suggested an association of high *TP73-AS1* expression with low-grade glioma histology [25], while its forced overexpression resulted in reduced proliferation, as well as induction of apoptosis in conventional GBM cell lines [26]. Finally, hypermethylation and low expression of *TP73-AS1* were found in GBM samples belonging to the less aggressive IDH and G-CIMP+ GBM subgroup^22^. Nevertheless, the clinical relevance or biological functions of *TP73-AS1* in GBM, and in particular, in gCSC are currently unknown.

Here, we show that the lncRNA *TP73-AS1* is clinically relevant in GBM, as high expression is associated with poor patient outcome in three independent, nonoverlapping primary GBM patient cohorts. Furthermore, *TP73-AS1* downregulation leads to loss of *ALDH1A1* expression and re-sensitizes gCSC to TMZ treatment. Together, our study underscores the importance of lncRNA-driven tumor biology in GBM and brings forth *TP73-AS1* as a promising prognostic biomarker and a therapeutic target in this fatal disease.

## Results

### TP73-AS1 is a GBM-associated lncRNA

**Fig. 1:**
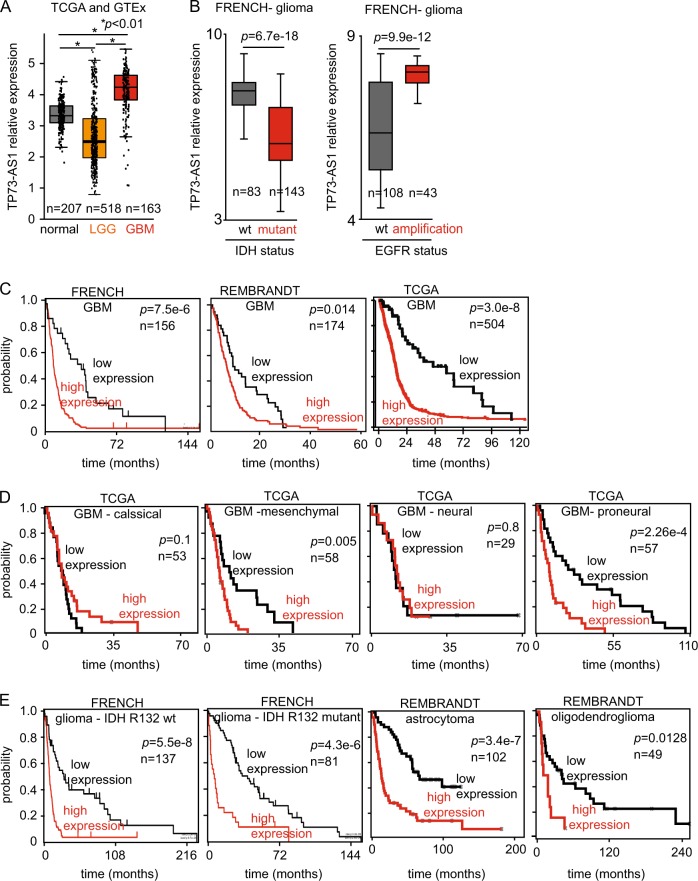
*TP73-AS1* is relevant in glioblastoma. **a** *TP73-AS1* expression in normal, LGG, and GBM tumor tissue. Normal tissue data were obtained from GTEx and tumor data from TCGA. Data were analyzed using GEPIA (gepia.cancer-pku.cn)^23^. **b** *TP73-AS1* expression in tumor samples with or without IDH1 R132 mutation. Data were analyzed using R2^65^. **c** Kaplan–Meier plots of patient outcome based on *TP73-AS1* expression. Plots were generated using http://www.betastasis.com/ and R2^65^. **d** Kaplan–Meier plots of patient outcome based on *TP73-AS1* expression in GBM. Plots were generated using http://www.betastasis.com/. **e** Kaplan–Meier plots of patient outcome based on *TP73-AS1* expression in glioma, stratified according to the annotated IDH mutation status and glioma subtype. Data, statistical evaluation, and visualization were obtained using the R2 website “R2: Genomics Analysis and Visualization Platform” (http://r2.amc.nl) and http://www.betastasis.com/

To assess whether *TP73-AS1* is clinically relevant in GBM, we used GEPIA (http://gepia.cancer-pku.cn/index.html) where GBM expression data, obtained from the TCGA, are compared with normal brain tissue data, obtained from GTEx, in a standardized manner^23^. *TP73-AS1* expression is significantly higher in primary GBM vs. normal brain tissue; however, it is lower in low-grade glioma (LGG) compared with normal tissue (Fig. 1a). Using R2, we analyzed the annotated FRENCH GBM cohort and found that the expression of *TP73-AS1* is associated with the more aggressive gliomas as its expression is lower in tumors carrying an IDH1 mutation, as compared with tumors with wild-type (wt) IDH1 (Fig. 1b) and is higher in EGFR-amplified glioma tumors (Fig. 1b), both of which are more aggressive gliomas.

Importantly, we demonstrate that high expression levels of *TP73-AS1* are associated with poor prognosis in three nonoverlapping, independent GBM cohorts (Fig. 1c). By analyzing TCGA survival and expression data, we found that high *TP73-AS1* expression is correlated with poor patient outcome in the “mesenchymal” and “proneural” subtypes but did not correlate with survival in the “classical” or “neural” subtypes (Fig. 1d). In glioma, high expression of *TP73-AS1* is significantly correlated with poor patient outcome in all tested cases, namely, IDH wt and mutant tumors, astrocytoma, and oligodendroglioma (Fig. 1e).

Collectively, these data suggest that *TP73-AS1* comprises a clinically relevant lncRNA, potentially contributing to the aggressive tumor biology of GBM.

### TP73-AS1 is linked to the stemness of gCSC

**Fig. 2:**
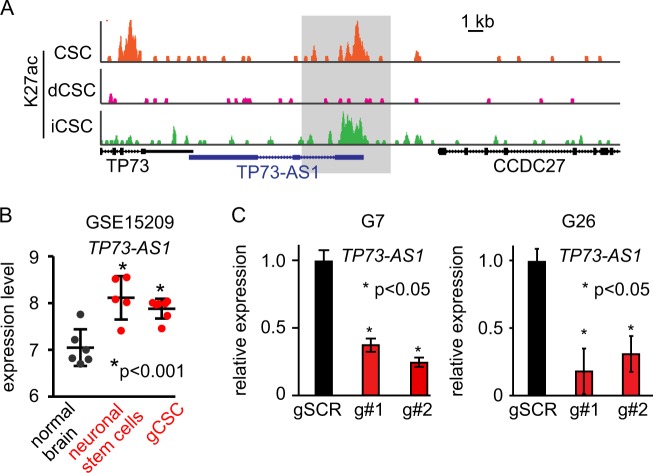
*TP73-AS1* kd does not induce cell death in gCSC. **a** Histone K27ac of the *TP73-AS1* locus in gCSC (CSC), differentiated gCSC (dCSC), and differentiated gCSC that had been reprogrammed to gCSC (iCSC). The putative promoter region of *TP73-AS1* is highlighted in gray. Data were obtained from graphs generated using http://www.broadinstitute.org/epigenomics/dataportal/clonePortals/Suva_Cell_2014.html. **b** The expression level of the *TP73-AS1* in normal brain tissue versus neuronal stem cells and gCSC. Data, statistical evaluation, and visualization were obtained using the R2 website “R2: Genomics Analysis and Visualization Platform” (http://r2.amc.nl). **c** Efficient knockdown of the lncRNA *TP73-AS1* in GBM tumor cells using CRISPRi. gCSC cells expressing a doxycycline-inducible dCAS9-KREB fusion protein and the indicated gRNAs targeting TP73-AS1 promoter region were generated. The indicated GBM tumor cell lines were engineered to express doxycyline-inducible dCAS9-KREB and gRNA targeting the TP73-AS1 promoter or scramble controls. Cells were induced with doxycycline for 10 days, after which the levels of *TP73-AS1* were measured using qRT-PCR. *p < 0.05; *n* = 3. dCAS9-KREB expression was induced after 10 days, and subsequently, the percentage of cell death induction was determined using Annexin V and PI staining and was measured by flow cytometry. Representative images are shown. The graph represents average ± SD; *n* = 3

A key aspect of transcriptional regulation of stemness factors occurs at the epigenetic level. In particular, Suvà et al. compared the epigenetic landscapes, as determined by histone K27 acetylation (K27ac) between gCSC, differentiated gCSC (dCSC), and differentiated CSC that were reprogrammed back to gCSC using a set of their four reprogramming factors (iCSC)^24^. Our analysis of their data revealed that K27ac patterns within *TP73-AS1* are similar to those of critical stemness factors in gCSC (Fig. 2a). Indeed, in gCSC, the *TP73-AS1* promoter is strongly K27ac-positive, indicating active transcription, while almost no K27ac was observed in differentiated gCSC. Upon reprogramming of dCSC (iCSC), the amount of K27ac within the *TP73-AS1* promoter increased again (Fig. 2a). These data indicate that the K27ac status of our candidate’s promoter is tightly linked to GBM cell “stemness” state, suggesting that *TP73-AS1* may constitute an important factor in gCSC.

We interrogated published data established with patient-derived gCSC growing under adherent conditions^4,25–27^. We found that *TP73-AS1* is highly expressed in gCSC and neuronal stem cells, as compared with the normal cortex (Fig. 2b). Due to the high expression of *TP73-AS1* in gCSC, we subsequently used a gene silencing approach in these cells to study the functions of *TP73-AS1*.

In order to examine the role of *TP73-AS1* in gCSC, we used two established gCSCs, G26 and G7^4,25^. As the biological function of some lncRNAs is tightly linked to their transcription^28–30^, we performed CRISPR inhibition (CRISPRi) as a gene knockdown (kd) approach^31,32^. In CRISPRi, a guide RNA (gRNA) targeting the region close to the transcriptional start site of a specific gene is expressed in a cell. The gRNA binds to a DNAse dead mutant CAS9 (dCAS9) fused to the transcriptional inhibitor domain KREB and the complex silences transcription of the targeted gene. We used a lentivirus approach to generate a stable gCSC, G26, and G7, expressing a doxycycline-inducible dCAS9-KREB^33^ in combination with either one of the two gRNAs targeting *TP73-AS1* (g#1 and g#2) or scramble controls (gSCR). Indeed, following doxycycline induction, qRT-PCR analysis showed significant reduction in *TP73-AS1* expression in the gCSC models expressing either one of the two gRNAs as compared with controls (Fig. 2c).

**Fig. 3:**
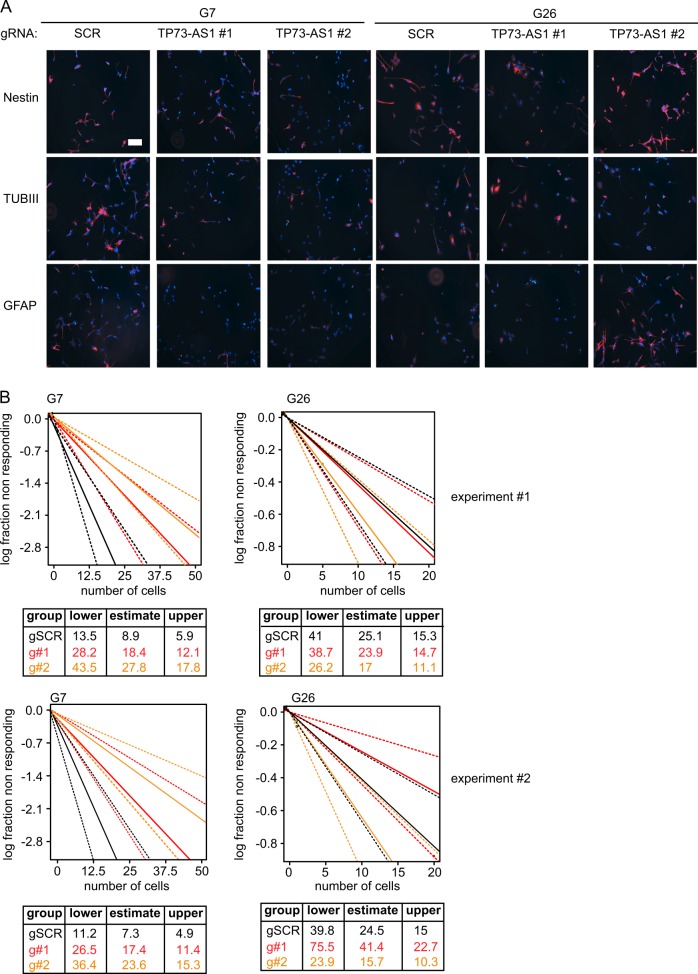
*TP73-AS1* promotes self-renewal in G7 but not in G26 gCSC. **a** gCSC cells expressing a doxycycline-inducible dCAS9-KREB fusion protein and the indicated gRNAs targeting TP73-AS1 promoter region were generated. dCAS9-KREB expression was induced for 10 days, after which the expression of the indicated stemness and differentiation markers was determined using immunofluorescence. Representative images are shown. Bar = 100 μM. **b** LDA assay measures the self-renewal capacity. dCAS9-KREB expression was induced for 10 days, after which the cells were plated in 96-well plates in the concentration of (1, 5, 10, and 20 cells per well). Spheres were counted manually 21 days after the induction. The estimate depicts the confidence interval for one per stem cell frequency. Data were analyzed using http://bioinf.wehi.edu.au/software/elda/

To determine whether *TP73-AS1* expression levels alter the stemness of gCSC, we assess the level of cellular differentiation upon *TP73-AS1* depletion in gCSC. We depleted *TP73-AS1* in gCSC for 10 days, and subsequently, we stained these cells for the established stemness (NESTIN) or differentiation markers (GFAP and TUBII)^4^ (Fig. 3a). Our results showed that *TP73-AS1* depletion did not result in consistent changes in the expression of the tested markers in the two tested gCSC models (Fig. 3a). To functionally test the self-renewal, an important stemness trait of *TP73-AS1*-depleted gCSC, we performed a limiting dilution assay (LDA), which measures the ability of low cell numbers to form a sphere^34^. To this end, we induced kd of *TP73-AS1* in gCSC for 10 days, plated cells in different dilutions in 96-well plates, and monitored sphere formation after 21 days (Fig. 3b). We found that *TP73-AS1* kd reduced the ability to form a sphere in G7 cells as compared with control cells (Fig. 3b). Nevertheless, this was not the case in G26 gCSC (Fig. 3b). These data suggest that *TP73-AS1* may act as a stemness factor in some gCSCs but not in all cases.

### Co-expression analysis reveals that TP73-AS1 expression affects TP73 expression inconsistently in gCSCs

The lncRNA *TP73-AS1* has an ~200-bp 3′ overlap with the 3′ untranslated region of an adjacent gene, *TP73* (p73) (Fig. 2a). As a member of the p53 family of transcription factors^35^, p73 has been implicated in several biological categories, in particular in cancer^36^, brain development^37^, and importantly, in the stemness of neuronal progenitor cells^38,39^ and invasiveness of GBM tumor cells^40^. Because many lncRNAs are known to directly regulate the expression of adjacent genes^28–30^, we investigated whether *TP73-AS1* regulates the expression of *TP73*. First, we tested whether silencing of *TP73-AS1* affected the expression of *TP73* in our gCSC models. However, we were not able to detect consistent changes in *TP73* expression. While in G7 cells, *TP73-AS1* kd led to a reduction of *TP73* expression, but the levels of *TP73* in G26 cells decreased upon silencing of *TP73-AS1* using g#1, while it increased in g#2 cells (SUP Fig. 1a).

Next, we asked whether the expression of *TP73-AS1* is correlated with that of *TP73* in expression data obtained from various (627) human cell lines and two primary GBM tumor datasets. We found that the expression of these two genes is not correlated (SUP 1b). We therefore conclude that it is unlikely that *TP73-AS1* directly regulates the expression of its neighboring gene*TP73*.

### TP73-AS1 promotes gCSC resistance to TMZ

Treatment with TMZ is the most common therapeutic approach used for GBM patients; therefore, it is critical to better understand the mechanisms underlying TMZ resistance in gCSC^41^. Because the expression of *TP73-AS1* is negatively correlated with patient survival, we asked whether *TP73-AS1* is involved in TMZ resistance in gCSC.

**Fig. 4:**
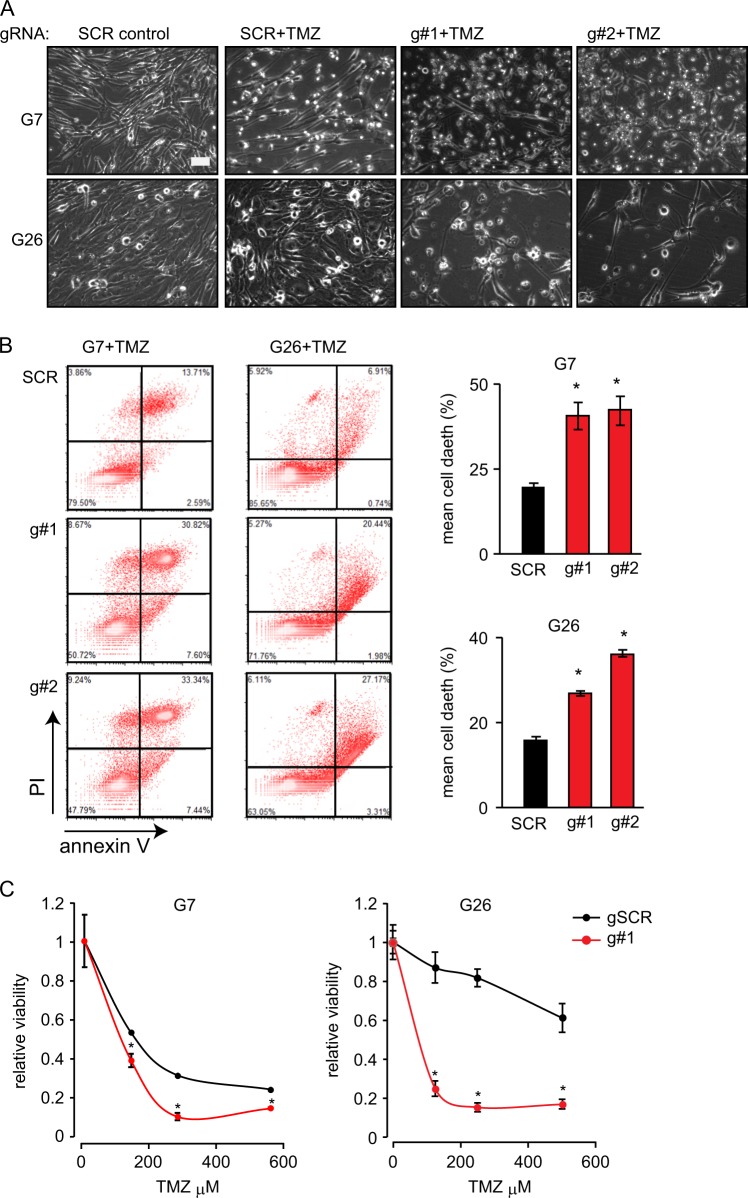
*TP73-AS1* promotes gCSC resistance to TMZ. **a** Representative images of *TP73-AS1* depleted and control gCSC cells treated with TMZ (500 µM) for 7 days. Images were recorded using a light microscope. **b** *TP73-AS1* depleted and control gCSC models were treated with TMZ (500 µM) for 7 days, after which the viability was determined using Annexin V/PI staining and flow cytometry. Representative images are shown. Bar = 50 μM; *p < 0.05; *n* = 3; average ± SD. **c** *TP73-AS1* depleted and control gCSC were treated with the indicated dose of TMZ for 7 days, after which viability was measured using crystal violet staining and a plate reader. Values represent the relative average OD; *n* ≥ 3; *p < 0.05 Student’s *t* test

Following the induction of *TP73-AS1* depletion for 10 days, we treated both gCSC models with TMZ for 7 days, which was sufficient to observe the morphological changes suggestive of increased cell death in the *TP73-AS1* kd cell culture (Fig. 4a). Subsequently, we quantified cell death induction in the TMZ-treated gCSC using annexin V/PI staining and FACS, and we found a significant increase in cell death in the *TP73-AS1*-depleted cells, as compared with control cells 5 days post treatment (Fig. 4b). Importantly, there was no difference in cell death between control and *TP73-AS1* kd cells under control conditions or 2 days post TMZ treatment (SUP Fig. 2a, b) indicating that *TP73-AS1* kd synergizes with TMZ to kill gCSC.

To confirm these findings, we measured the viability of G7 and G26 gCSC treated with an escalating dose of TMZ for 5 days and found that the kd of *TP73-AS1* in both gCSCs led to an increased sensitivity to TMZ (Fig. 4c).

We investigated the impact of *TP73-AS1* on gCSC proliferation using a BrdU assay. We found that there was no significant difference between *TP73-AS1* kd and control cells under resting conditions, and that proliferation was inhibited in all cases 2 days following TMZ treatment (SUP Fig. 3a). In addition, we measured reactive oxygen species (ROS) in *TP73-AS1* depleted and control gCSC cells, using DCFDA (chloromethyl-2′,7′-dichlorofluorescein diacetate) and FACS, and found that in G7 cells, there were no differences in ROS levels in all tested conditions. In contrast, *TP73-AS1* depletion resulted in increased ROS levels following 2 days of TMZ treatment and in non-treated G26 cells (SUP Fig. 3b).

These data indicate that *TP73-AS1* contributes to TMZ resistance in gCSC and that the protective functions of *TP73-AS1* are not linked to regulation of proliferation. Furthermore, *TP73-AS1* protective functions in gCSC may be due to curbing ROS levels in a cell-line-specific manner.

### TP73-AS1 interacts with multiple metabolic pathways in TMZ-treated gCSC

O-6-Methylguanine-DNA methyltransferase (MGMT) is known to repair DNA damage caused by alkylating agents, including TMZ, and it is regulated in GBM at the level of promotor methylation^42^, and MGMT methylation status is linked to the response to TMZ in patients. We asked what is the status of the promotor of MGMT in our gCSC using published data^43–45^ and found that the promoter is methylated in both cell lines (SUP Fig. 3c), suggesting that the increased sensitivity of the *TP73-AS1* kd cells is not likely to be related to MGMT methylation.

To learn more about the cellular pathways interacting with *TP73-AS1*, we compared the transcriptome of *TP73-AS1* kd vs. control G7 and G26 gCSC upon TMZ treatment and control conditions using RNAseq. According to our principal component analysis (PCA), we found that all three biological replicates from each experimental condition grouped together indicate that the major differences between samples are due to the biological perturbations caused by the knockdown of *TP73-AS1* or TMZ treatment (SUP Fig. 4a).

Next, we asked which of the transcripts are affected by *TP73-AS1* kd in each cell line upon TMZ treatment or in control conditions (SUP Table 1). To this end, we clustered the genes, which are significantly (DeSeq2 with a cutoff of the adjusted *p* value < 0.05) up- and downregulated upon *TP73-AS1* kd. We found that there were profound transcriptional changes affecting over 2000 genes that were significantly changed in each experimental condition. For validation, we chose four transcripts, measured their expression using qRT-PCR, and found that their expression pattern matched the pattern found in the RNAseq data (Sup Fig. 5 and Fig. 6a).

**Fig. 5:**
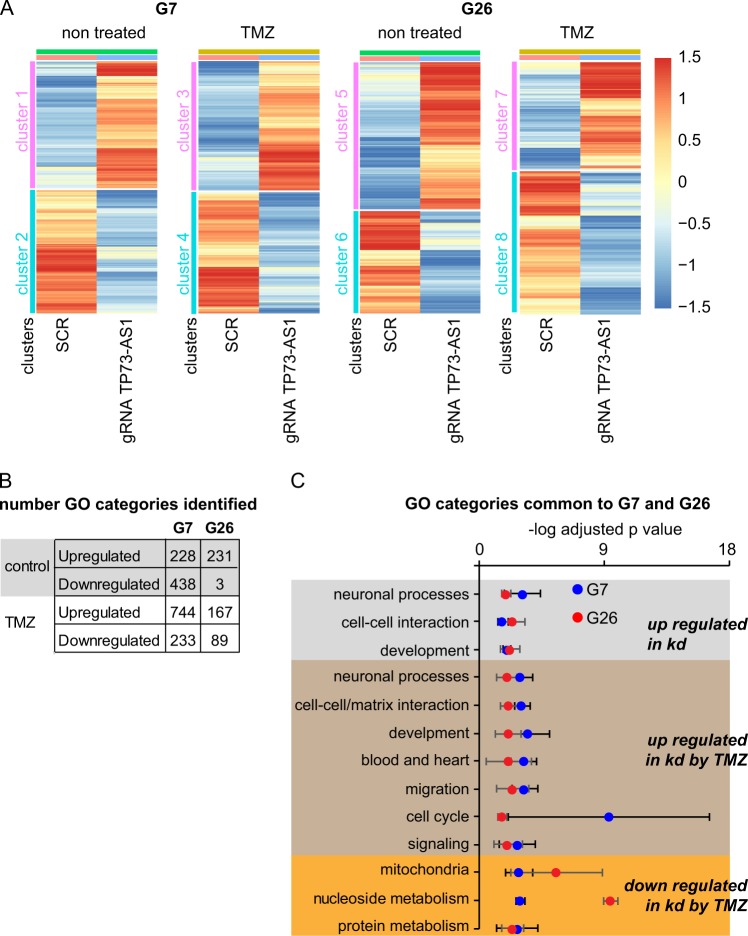
Transcriptional landscape upon *TP73-AS1* depletion in gCSCs. **a** Heat map depicting transcripts whose expression is affected by *TP73-AS1* kd. Red represents upregulation and blue represents downregulation. **b** Transcripts affected by *TP73-AS1* kd in each cell line and conditions were analyzed to identify significantly affected GO categories. The number of GO categories identified in each cell line, condition, and direction of gene expression change (upregulation or downregulation by *TP73-AS1* kd) are shown. **c** Adjusted *p* values (average ± SD) of GO categories related to the indicated biological functions are shown. Note that there were no common categories in the list of genes that were downregulated in the kd cells. See Table S1 for a complete list

The *TP73-AS1*-dependent transcriptome perturbation indicates that *TP73-AS1* comprises a major biological regulator in these cells (Fig. 5a). We therefore asked if there was a significant enrichment for particular biological pathways upon *TP73-AS1* kd and found that there were hundreds of gene ontology (GO) pathways affected (Fig. 5b). In both G7 and G26 cells, *TP73-AS1* kd led to an upregulation of genes enriched for GO categories related to neuronal development and function, in addition to other developmental pathways and cell–cell communication pathways (Fig. 5c and SUP Table 2).

Under TMZ treatment, genes belonging to neuronal differentiation and function were also upregulated in *TP73-AS1* kd cells in both gCSC models (Fig. 5c and SUP Table 2). Interestingly, transcripts related to metabolism, mitochondria, and nucleotide metabolism in particular, were downregulated upon *TP73-AS1* kd in both cell lines (Fig. 5c and Table 2). These findings may explain how *TP73-AS1* kd leads to increased sensitivity to the alkylating agent TMZ in gCSC, as chemotherapy resistance is tightly linked to cellular metabolism^46^. Together, our transcriptomic analysis reveals that *TP73-AS1* is a major regulator in gCSC by orchestrating multiple cellular pathways, including neuronal and metabolic pathways, providing protection against TMZ.

### TP73-AS1 promotes the expression of ALDH1A1

**Fig. 6:**
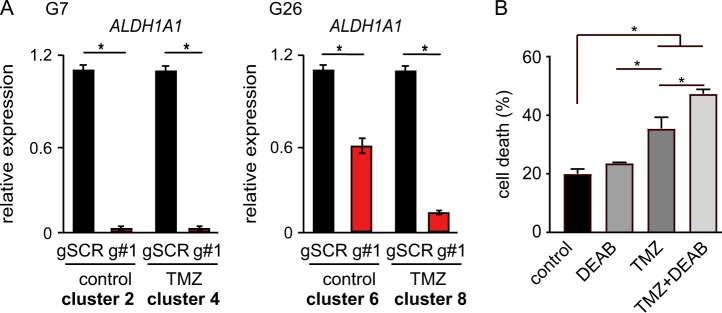
*TP73-AS1* depletion leads to reduced *ALDH1A1* expression. **a** The indicated gCSC lines were treated with TMZ and the levels of *ALDH1A1* were measured using qRT-PCR. The results matched the trends found in RNAseq experiments, as indicated by the RNAseq group each condition belongs to. **b** G7 cells were treated with TMZ (300 μM), DEAB (200 μM), or a combination of the two compounds for 5 days, after which cell death was measured using PI and FACS. Average values and SEM are shown. *n* = 3; *p < 0.05 ANOVA

One of the transcripts found to be downregulated in the two *TP73-AS1* kd cell lines under TMZ treatment and control conditions was *ALDH1A1* (Fig. 6a), which encodes the aldehyde dehydrogenase 1 family member A1 (ALDH1A1) protein. This protein family is known for their ability to do detoxification of endogenous and exogenous aldehyde substrates through NAD(P)+ oxidation^47^. Moreover, ALDH1A1 is a known marker of stem cells and cancer stem cells, and plays a crucial role in chemoresistance in several cancer types^48,49^ and specifically in GBM^50^.

To test if *ALDH1A1* downregulation explains, at least in part, the protective functions of *TP73-AS1* in gCSC, we treated gCSC with the TMZ (300 μM) in the presence or absence of the ALDH1 inhibitor, DEAB (200 μM), for 5 days, after which we measured cell death using PI and FACS (Fig. 6b). We found that DEAB increased the sensitivity of G7 cells to TMZ, confirming the protective functions of ALDH1 in gCSC and providing support to the model where *TP73-AS1* enhances the resistance of gCSC to TMZ by promoting the expression of *ALDH1A1*.

## Discussion

The biological role and the clinical relevance of *TP73-AS1* in GBM are only emerging. Contradictory results were recently published, suggesting either a tumor promoting^51,52^ or suppressing^53^ function of *TP73-AS1* in GBM. Specifically, a recent study demonstrated that overexpression of *TP73-AS1* in U251 GBM tumor cells resulted in reduced proliferation and increased cell death^53^. Furthermore, the same study suggested that the expression of *TP73-AS1* was higher in low-grade vs. high-grade gliomas. However, other studies showed that high expression of *TP73-AS1* in peritumoral brain edema was correlated with poor patient outcome and that depletion of *TP73-AS1* in GBM tumor cell lines results in reduced proliferation and migration^52^. To clarify the clinical relevance of the lncRNA *TP73-AS1* in GBM, we interrogated three GBM patient cohorts and found that high *TP73-AS1* expression was significantly correlated with poor patient outcome. This is in accordance with the finding that the expression of *TP73-AS1* is high in GBM and low in LGG. Furthermore, *TP73-AS1* is highly expressed in IDH1 wt vs. mutant tumors, the former being more aggressive than the latter.

To elucidate the biological functions of *TP73-AS1*, we utilized gCSC models, which are the most relevant in vitro models for studying GBM biology^4^. These gCSC models generate tumors upon orthotopic implantation into brains of immunocompromised mice, which most closely recapitulate the initial patient-derived tumor^4,25^. Using CRISPRi as a gene silencing approach and gCSC, we found that *TP73-AS1* depletion does not affect neither proliferation nor gCSC survival. It may be that *TP73-AS1* functions in a cell context-dependent manner and may promote or inhibit proliferation in standard GBM cell lines as opposed to gCSC.

Notably, the epigenetic profile of the *TP73-AS1* promoter in differentiated, reprogrammed, and untreated gCSCs is similar to that found in the promoter of the established stemness factors^24^. Using a functional assay (LDA), we found a significant effect on self-renewal upon *TP73-AS1* silencing in one of our tested gCSC, G7 but not in G26. These differences may be related to the type of tumor from which these cells were generated, the specific cell line biology, or compensatory mechanisms upon *TP73-AS1* kd. Indeed, there are notable differences in key stemness factors expressed in these cells^25^. Nevertheless, we found that *TP73-AS1* kd led to an upregulation of transcripts associated with neuronal differentiation in both models.

The current standard of care for GBM patients includes surgery, followed by radiation combined with chemotherapy^54^. Temozolomide is the major chemotherapeutic agent used for GBM patients. Hence, our finding that *TP73-AS1* affected TMZ resistance in gCSC is of immediate clinical relevance. Specifically, we showed that *TP73-AS1* silencing led to a significantly increased sensitivity to TMZ in two gCSC models. Despite an aggressive surgical approach and aggressive chemotherapy, the prognosis of GBM patients remains dismal^54^. Cancer stem cells are especially chemoresistant^55,56^ and are characterized by high expression of DNA repair proteins and efflux pumps^57,58^. Our data may explain the tight association existing between high *TP73-AS1* expression and poor patient outcome in GBM and strongly suggest that *TP73-AS1* exhibits a chemoprotective role in GBM tumors.

The mechanism by which *TP73-AS1* protects gCSC from TMZ remains currently unknown. Because in some cases, lncRNAs function by regulating their gene neighbors^28–30^, we first tested whether *TP73-AS1* may regulate the expression of its gene neighbor, *TP73*. We found that this is not the case, as there is no correlation in expression between the two genes in G26, in a collection of mammalian cell lines and in two independent GBM cohorts. However, it is possible that there is an indirect promotion of *TP73* expression by *TP73-AS1* in specific cellular contexts, as is the case in G7 cells. These data are consistent with the known functions of TP73 as a stemness factor in brain stem cells^38,39^ and the finding that in G7 gCSC, but not G26 gCSC, *TP73-AS1* promotes self-renewal (Fig. 3b).

Using expression profiling, we found that *TP73-AS1* kd and TMZ treatment resulted in reduced expression of transcripts related to several metabolic pathways, including mitochondria biology and nucleoside metabolism. While gCSCs can adapt to different nutrients found in their environment, they are highly dependent on the mitochondria for generating energy^59^. Furthermore, recent findings suggest that combining mitochondrial toxins with TMZ may be beneficial in eradicating gCSC^60^. Importantly, a recent CRISPR activation screen, aimed at identifying both protein-coding and lncRNA genes promoting drug resistance in acute myeloid leukemia tumor cells, found that lncRNA-protecting tumor cells from chemotherapy were often neighbors and regulators of metabolism-related genes, highlighting the importance of lncRNA regulation of the expression of metabolic genes in drug resistance^46^. It is therefore tempting to speculate that *TP73-AS1* protects gCSC from TMZ by promoting mitochondrial and other metabolic pathways.

Our RNAseq data and validation showed that one of the transcripts that is downregulated under TMZ treatment and control conditions in the two *TP73-AS1* kd gCSCs is *ALDH1A1* (sup table 1 and sup Fig. 6), which encodes the aldehyde dehydrogenase 1 family member A1 (ALDH1A1) protein. ALDH1A1 is known to be a marker of cancer stem cells^47,61^ and importantly, is known to promote drug resistance in cancer^62^, cancer stem cells, and specifically in gCSC^61,63^. Using the ALDH1 inhibitor, DEAB, we confirmed that also in the case of the gCSC used in this study, ALDH1 inhibition leads to enhanced TMZ sensitivity, providing support to the premise that the increased TMZ sensitivity of *TP73-AS1*-depleted cells is linked to reduced *ALDH1A1* expression. The mechanism of ALDH1A1-mediated therapy resistance is still not completely understood, but was proposed to be linked to reduction of oxidative stress caused by the chemotherapy^62^. We therefore propose that by promoting ALDH1A1 expression in gCSC, *TP73-AS1* reduces their sensitivity to chemotherapy.

In conclusion, we uncovered that *TP73-AS1* comprises a clinically relevant lncRNA conferring TMZ resistance in gCSC, and may therefore serve as an important predictive biomarker and a therapeutic target in a currently fatal disease.

## Materials and methods

### Cell culture

G26 and G7 were a kind gift from Steven Pollard. Cells were grown on plates coated with Laminin (Sigma-Aldrich, L2020), as detailed in ref. ^4^.

### Immunofluorescence

Cells were cultured and differentiated in a 96-well plate covered with poly-L-ornithine hydrobromide (Sigma-Aldrich; P3655) and Laminin (10% in PBS). Cells were grown overnight, after which they were fixed with 4% paraformaldehyde (paraformaldehyde 16%, Alfa Aesar) in PBS for 30 min and permeabilized with permeabilization buffer (0.5% Triton X-100 (Sigma-Aldrich) in PBS). The cells were washed with washing solution (25% BSA (bovine serum albumin), Sigma-Aldrich) and 0.1% Tween-20 (Sigma-Aldrich, P9416; PBS). The cells were blocked for 1 h with 10% goat serum in wash buffer and subsequently in washing solution. The blocking buffer was removed and the cells were labeled with primary antibodies (anti-nestin 1:100; Abcam (10C2); anti-TUBIII 1:75 (Merck (MAB1637); anti-GFAP 1:100 (Santa Cruz (2E1)) diluted in blocking buffer and incubated overnight at 4 °C. The cells were incubated at room temperature (RT) for 30 min and then washed using washing solution. A secondary antibody (ENCO, Alexa 549) was added (1:200 in blocking solution) for 1 h at RT. The cells were washed with the washing solution and stained with DAPI for 1 min. The cells were washed three times with DDW. Images were taken using a fluorescent microscope.

### Cell proliferation assessment with 5-bromo-2-deoxyuridine (BrdU)

The BrdU proliferation assay was performed using BrdU Cell Proliferation Assay Kit (Biovision Incorporated) following the manufacturer’s instructions. Cells were cultured in 96-well plates covered with poly-L-ornithine and laminin (10% in PBS) overnight, after which the cells were labeled with 5-bromo-2-deoxyuridine (BrdU) for 3 h and stained. Cells were visualized under a fluorescent microscope and the number of BrdU-positive cells and the total cell number were determined in each field.

### RNA extraction, cDNA synthesis, and quantitative reverse transcription polymerase chain reaction (qRT-PCR)

The RNA from cultured cells was purified using the PureLink RNa Mini Kit (Thermo Fisher Scientific). From a starting amount of 100–200 ng of RNA, cDNA was generated using the cDNA synthesis kit (E6300S, BioLabs). Briefly, 20 µl of the cDNA synthesis reaction was subjected to the following conditions: 5 min at 25 °C, 60 min at 72 °C, and 5 min at 96 °C. cDNA is synthesized in vitro, from an mRNA template using the enzyme reverse transcriptase, resulting in single-stranded cDNA production. qPCR was carried out on the produced cDNA to allow for detection of mRNA expression levels in the cells of interest. The PCR conditions were as follows: initialization step at 95 °C for 10 min, 46 cycles of the denaturation step at 60 °C for 30 s, annealing step at 60 °C for 30 s, and elongation step at 72 °C for 1 s, followed by the final elongation at 40 °C for 10 min to ensure that any remaining single-stranded DNA is fully extended. Most of the forward and the reverse primers were designed to span different exons whenever possible. All primers were purchased from Integrated DNA Technologies (IDT). qRT-PCR was performed using an iCycler (Bio-Rad Laboratories) with a threshold cycle number determined with the use of iCycler software version. Reactions were performed in triplicate and threshold cycle numbers were averaged. The results were normalized to L32 and POL2.

### RT-PCR primers used in this study

TP73-AS1: probe #29, primer1 ctccggacactgtgttttctc, primer2 tcttttaaggcggccatatc; P73: probe #60, primer1 cacgtttgagcacctctgg, primer2 cgcccaccacctcattatt; L32: probe #33, primer1 gcacactgactacagccttga, primer2 tacccaggtttggaggtgtg; ALDH1A1 #14: primer1 tttggtggattcaagatgtctg, primer2 cactgtgactgttttgacctctg; FNDC5 #8: primer1 taccaaaacaccccttctgg, primer2 tcttcctgtccgtggtgaat; ZNF536 #22: primer1 gagtcccagtcggtgagc, primer2 gctctcctcggtgacgttag; WNT5A #11: primer1 ttctggctccacttgttgct, primer2 gccaaagccactaggaagaac.

### Plasmids

psPAX2 was a gift from Didier Trono (Addgene plasmid # 12260). pMD2.G was a gift from Didier Trono (Addgene plasmid # 12259). pHAGE TRE dCas9-KRAB was a gift from Rene Maehr and Scot Wolfe (Addgene plasmid # 50917). pLKO.1-puro U6 sgRNA BfuAI large stuffer was a gift from Scot Wolfe (Addgene plasmid # 52628). We used pLKO.1-puro U6 sgRNA BfuAI large stuffer to clone our gRNA sequences according to the instructions available on the addgene page. The sequences used were as follows:

scramble/control: ACCGCGCCAAACGTGCCCTGACGG; TP73-AS1 g#1: GCAGTCGGGGCTGACGGCGG; TP73-AS1 g#2: CCTAGATGGGAGCCGGGGAT.

### Generation of stable cell lines for gene knockdown

Lentiviruses were generated using our standard protocol in 293T cells, as detailed in HACE1 reduces oxidative stress and mutant Huntingtin toxicity by promoting the NRF2 response^64^. Cells were grown in DMEM, 10% FBS, and transfected using CalFectin (Signagen) and plasmids at a ratio of 1:2:3 (PAX2; pMD2.G; transfer vector). Media was changed after 24 h and the viruses were collected after 48 h. The collected viruses were stored at −80 °C. G7 and G26 cells were grown to 80% confluence on 6-cm dishes, then infected with a vector ratio of 1:10 (cas9, gRNA), and left for 24 h in a 37 °C incubator. Following infection, the lentivirus-containing medium was removed and replaced by fresh medium and incubated at 37 °C with the selection antibiotic using puromycin (1 μg/ml) and G418 (1500 μg/ml).

### Gene knockdown

The depletion of the *TP73-AS1* was done using the CRISPRi method combined with viral infection. gRNA were designed to target the promoter region of the *TP73-AS1* gene. Knockdown induction was done using doxycycline at a concentration of 2 µg/ml (Sigma-Aldrich, D3072). Gene knockdown was measured using qRT-PCR.

### LDA-sphere formation assay

Cells are plated into a 96-well plate over a range of densities from high to low (number of cells/well = 1, 5, 10, and 20). The cells were allowed to grow for 21 days and form spheres. To determine the probability of sphere formation, the wells were scored for the presence or absence of sphere growth. The natural log fraction of the sphere-negative wells was plotted on a linear scale versus the density (cells/well). The frequency of sphere-forming cells in a particular cell type was determined using the ELDA web tool at http://bioinf.wehi.edu.au/software/elda.

### TMZ treatment

The kd of *TP73-AS1* was induced by addition of doxycycline for 10 days. The cells were plated in 12-well plates and TMZ (Sigma-Aldrich, T2577) or DMSO was added. Seven to ten days post treatment, the cells were washed and trichloroacetic acid (TCA) was added for an hour at 4 °C. The plates were washed and air dried. Crystal Violet (C0075, Sigma-Aldrich) was added for half an hour and additional rinsing was conducted. After dehydration, 10% acetic acid was added and the optical density was measured at 570 nm. The results were normalized to scramble gRNA.

### FACS

Cell apoptosis was determined using flow cytometry. Cells were harvested 10 days post doxycycline treatment using Accutase (Biological Industries, Israel) and the cells were treated with TMZ for 7 days and harvested later. The cells were stained using an apoptosis detection kit (Miltenyi Biotec). The cells were resuspended in binding buffer, and were then stained with Annexin V FITC and propidium iodide (PI). The cells were analyzed using a flow cytometer (Sysmex) equipped with FCS Express software (De Novo Software) according to the manufacturer’s instructions. For the DEAB treatments, G7 cells were treated with 200 μM of DEAB (D86256, Sigma-Aldrich), an ALDH1A1 inhibitor, and with 300 μM of TMZ for 5 days, after which cell death was measured using PI staining and FACS.

### ROS measurement

Cell quantitation of ROS was determined using flow cytometry. Cells were harvested 10 days post doxycycline treatment using Accutase (Biological Industries, Israel) and the cells were treated with TMZ for 2 days and harvested later. The cells were stained using a ROS detection kit (D6883, Sigma-Aldrich). The cells were treated with DCFDA for 1 h, and then harvested and washed with PBS. The cells were analyzed using a flow cytometer (Sysmex) equipped with FCS Express software (De Novo Software) according to the manufacturer’s instructions.

### RNAseq

Libraries were prepared using QuantSeq 3′ mRNA-Seq Library Prep Kit FWD for Illumina (Lexogene) according to the manufacturer’s protocol. Samples were pooled and sequencing was performed using Nextseq5000 using all four lanes.

Thirty-three samples were sequenced in the present study. These are composed of three biological replicates of G7 cells expressing gRNA SCR (scrambled) and gRNA#1 in TMZ treated and untreated conditions and three biological replicates of G26 cells expressing gRNA SCR, gRNA#1, and gRNA#2 under TMZ treatment and non-treated conditions. Sequencing resulted in an average of 9,655,276 reads per sample with an average sequence length of 86 bp. Raw reads were then trimmed for sequencing adaptors, poly-A, as well as poor-quality bases using Trim galore (v0.4.2; https://www.bioinformatics.babraham.ac.uk/projects/trim_galore/) and Cutadapt (v1.12.1; github.com/easybuilders/easybuild-easyconfigs/tree/master/easybuild/easyconfigs/c/cutadapt). After QC, an average of 9,561,457 reads per sample remained for downstream analysis with an average sequence length of 78 bp. Then mapping and gene-level read count estimation were performed using STAR (v2.5.3a) and RSEM (v1.2.31), respectively, against the human reference genome (GRCh38). The DeSeq2 R package was used to normalize gene counts and perform differential gene expression, followed by clustering and visualizations using the DeSeq2 VSD function (Variance Stabilizing Transformation). Clustering analysis was performed by employing a hierarchical clustering method (using Pearson correlation clustering and “ward.D2” agglomeration method), while the number of clusters were assessed using the “eclust” function from the “factoextra” R package. The “clusterprofiler” R package was used for Gene Ontology enrichment analysis, while gene annotation was retrieved from the “org.Hs.eg.db” R package. PCA was generated for the normalized count data using the plotPCA function from the DeSeq2 R package using the top 500 variable genes.

### Cell viability measurements using crystal violet staining

Cells were grown in a medium containing antibiotic selection. KD was induced with doxycycline for 10 days, after which the cells were replated into 24-well plates in the presence or absence of TMZ (Sigma-Aldrich, T2577). One week post treatment, the cells were washed with trichloroacetic acid (TCA) for an hour at 4 °C. The plates were washed and air dried. Crystal Violet (C0075, Sigma-Aldrich) was added for half an hour and additional rinsing took place. Following dehydration, 10% acetic acid was added and the OD was measured at 590 nm. The results were normalized to scramble gRNA.

## Supplementary information

Supplemental material

SUP table 1

SUP table 2

SUP Figure 1

SUP Figure 2

SUP Figure 3

SUP Figure 4

SUP Figure 5
