# Supplementary material for: The lncRNA TP73-AS1 is linked to aggressiveness in glioblastoma and promotes temozolomide resistance in glioblastoma cancer stem cells
Source: Cell Death Dis. 2019 Mar 13;10(3):246. doi: 10.1038/s41419-019-1477-5 (PMC6416247; doi:10.1038/s41419-019-1477-5)
Supplement: Supplementary file 1 — Supplemental material [file 41419_2019_1477_MOESM1_ESM.docx]

**Supplementary material**

***The lncRNA TP73-AS1 promotes temozolomide resistance in glioblastoma cancer stem cells***

Gal Mazor^1^, Liron Levin^2^, Daniel Picard^3,4,5^, Ulvi Ahmadov^3,4,5^, Helena Carén^6^, Arndt Borkhardt^4^, Guido Reifenberger^5^, Gabriel Leprivier^4^, Marc Remke^3,4,5^, Barak Rotblat^1^*

^1^ Department of Life Sciences, Ben-Gurion University of the Negev, Beer-Sheva, Israel

^2^ Bioinformatics Core Facility, National Institute for Biotechnology in the Negev, Ben-Gurion University of the Negev, Beer-Sheva, Israel

^3^ Department of Pediatric Neuro-Oncogenomics, German Cancer Research Center (DKFZ), Heidelberg, Germany, and German Cancer Consortium (DKTK), partner site Essen/Düsseldorf, Germany

^4^ Department of Pediatric Oncology, Hematology, and Clinical Immunology, Medical Faculty, University Hospital Düsseldorf, Düsseldorf, Germany

^5^ Institute of Neuropathology, Medical Faculty, University Hospital Düsseldorf, Düsseldorf, Germany

^6^ Sahlgrenska Cancer Center, Gothenburg, Sweden

* corresponding author rotblat@bgu.ac.il

p-values are provided. Sheared GO terms between tests are also summarized.

**Figure legends**

**SUP 1. *TP73-AS1* expression does not correlate with *TP73*.** **A.** RNA levels of the *TP73* and *TP73-AS1* were measured in the indicated gCSC induced to express dCAS9-KREB for 10 days with either one of two gRNA targeting *TP73-AS1* or scrambled control. n=3; average ± SD. **B.** Correlation between the mRNA expression of *TP73* and *TP73-AS1* in cell lines and GBM tumors were tested using the indicated datasets. Correlation graphs were generated using R2 website (http://r2.amc.nl). R and p values are shown.

**SUP 2.** ***TP73-AS1* depletion does not result in altered cell death of gCSC at two days post TMZ treatment.** Cell death was measured using annexin V/PI staining and FACS assay. **A.** Cell death measurements of indicated non-treated gCSC expressing the indicated gRNA. Typical FACS images are shown. n=3; Differences are non-significant in student’s t-test. **B.** The indicated gCSC expressing the indicated gRNA were treated with TMZ for two days after which death was measured. Typical FACS images are shown. n=3; Differences are non-significant in student’s t-test.

**SUP 3. Proliferation and ROS levels in *TP73-AS1* kd gCSC treated with TMZ.** gCSC were treated with TMZ for two days or left untreated. **A.** Cell proliferation of the indicated cell lines was measured using a BrdU assay. Typical microscopy images are shown. n=3; Note, no BrdU positive cells were found in the TMZ treated samples. **B.** ROS levels in the indicated cell lines were measured using CM-DCFDA. n=3; p<0.05. **C.** The methylation status of the MGMT promoter was predicted with the R package MGMT-STP27 [1]–[3].

**SUP 4. Transcriptional profiling of gCSC following *TP73-AS1* kd using RNAseq.** In G7 data we used gSCR and g#1. In G26 we used gSCR and grouped together g#1 and g#2 for analysis of *TP73-AS1* kd. Each data point represents three biological replicates. **A.** PCA plot of *TP73-AS1* kd and control G7 and G26 gCSC treated with TMZ or left untreated. **B.** Clusters of transcripts affected by *TP73-AS1* kd in gCSC treated with TMZ or controls. Numbers represent the number of genes in each cluster, the gray area around the line indicate 0.95 confidence interval.

**SUP 5. Validation of RNAseq findings using qRT-PCR.** The expression of the indicated genes in the indicated cell lines under TMZ treatment or control conditions was measured using qRT-PCR. The cluster to which the tested transcripts belongs to in the RNAseq analysis (as in SUP Fig. 3b) are indicated in bold. n=3; p<0.05

**Supplementary tables**

**Table 1. Differentially expressed genes information:**

A separate sheet is available for each statistical test performed in this study. For each cell type two test were performed *TP73-AS1* kd vs control in untreated and in TMZ treated cells. A list of differentially expressed genes along with their average normalized counts standardized, log2 fold change, adjusted p-values, cluster assignment as well as gene annotation are provided.

**Table 2. Gene Ontology terms enriched in differentially expressed genes:**

A separate sheet is available for each statistical test performed in this study as in Table 1. For each test significantly enriched GO terms in the different genes clusters are provided. Information about the GO term as well as its cluster and background gene ratio, genes id (ENTREZ id), native and adjusted

**References**

[1] M. E. Hegi *et al.*, “*MGMT* Gene Silencing and Benefit from Temozolomide in Glioblastoma,” *N. Engl. J. Med.*, vol. 352, no. 10, pp. 997–1003, Mar. 2005.

[2] H. Carén *et al.*, “Glioblastoma stem cells respond to differentiation cues but fail to undergo commitment and terminal cell-cycle arrest,” *Stem Cell Reports*, vol. 5, no. 5, pp. 829–842, 2015.

[3] P. Bady *et al.*, “MGMT methylation analysis of glioblastoma on the Infinium methylation BeadChip identifies two distinct CpG regions associated with gene silencing and outcome, yielding a prediction model for comparisons across datasets, tumor grades, and CIMP-status,” *Acta Neuropathol.*, vol. 124, no. 4, pp. 547–560, Oct. 2012.
