# Supplementary figures and images for: The lncRNA TP73-AS1 is linked to aggressiveness in glioblastoma and promotes temozolomide resistance in glioblastoma cancer stem cells
Source: Cell Death Dis. 2019 Mar 13;10(3):246. doi: 10.1038/s41419-019-1477-5 (PMC6416247; doi:10.1038/s41419-019-1477-5)

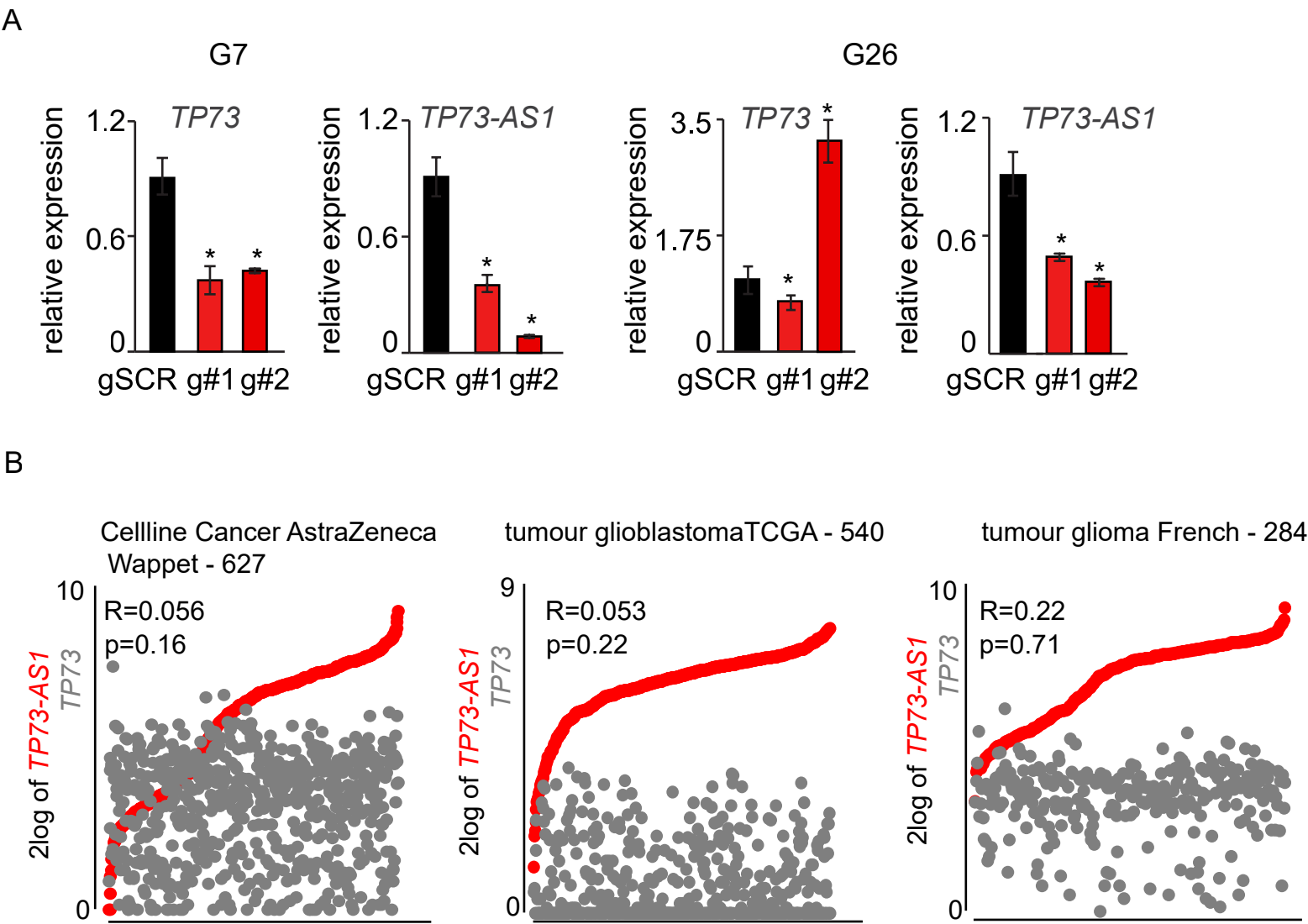

Supplement: Supplementary file 4 — SUP Figure 1 [file 41419_2019_1477_MOESM4_ESM.pdf]

A

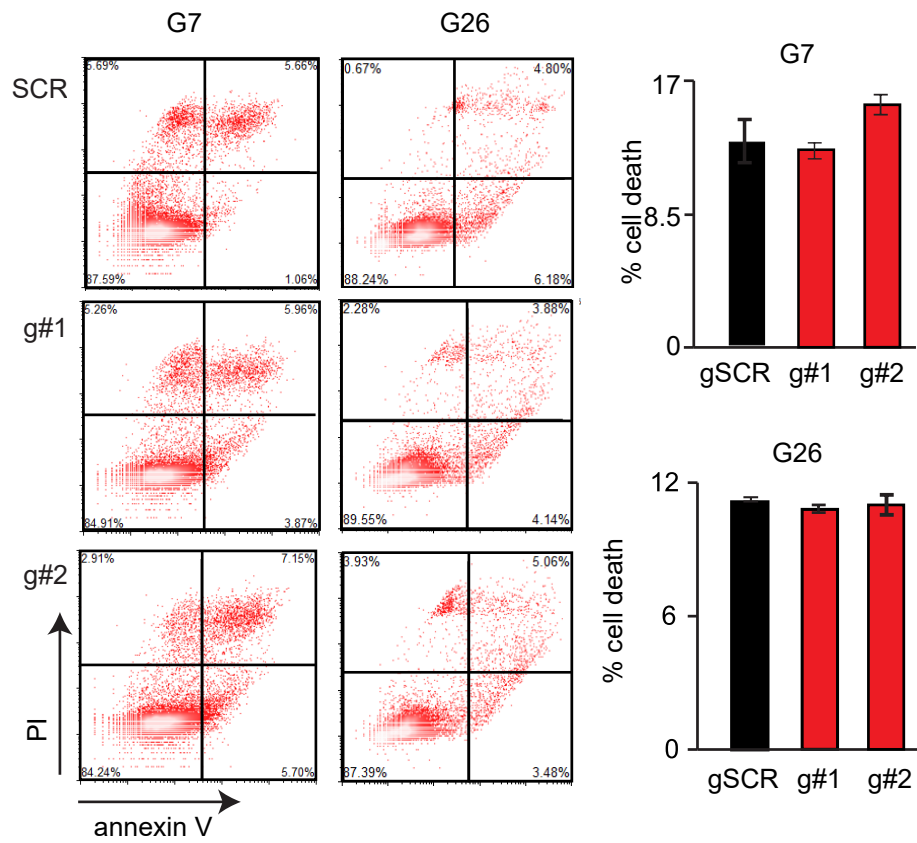

B

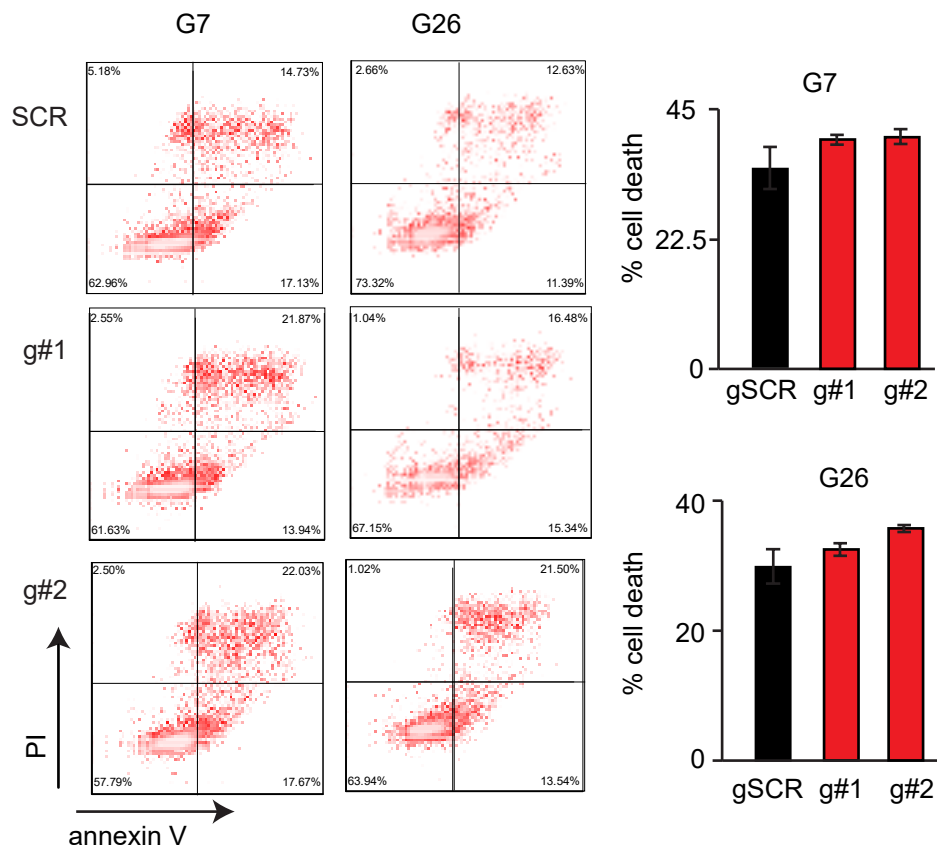

Supplement: Supplementary file 5 — SUP Figure 2 [file 41419_2019_1477_MOESM5_ESM.pdf]

A

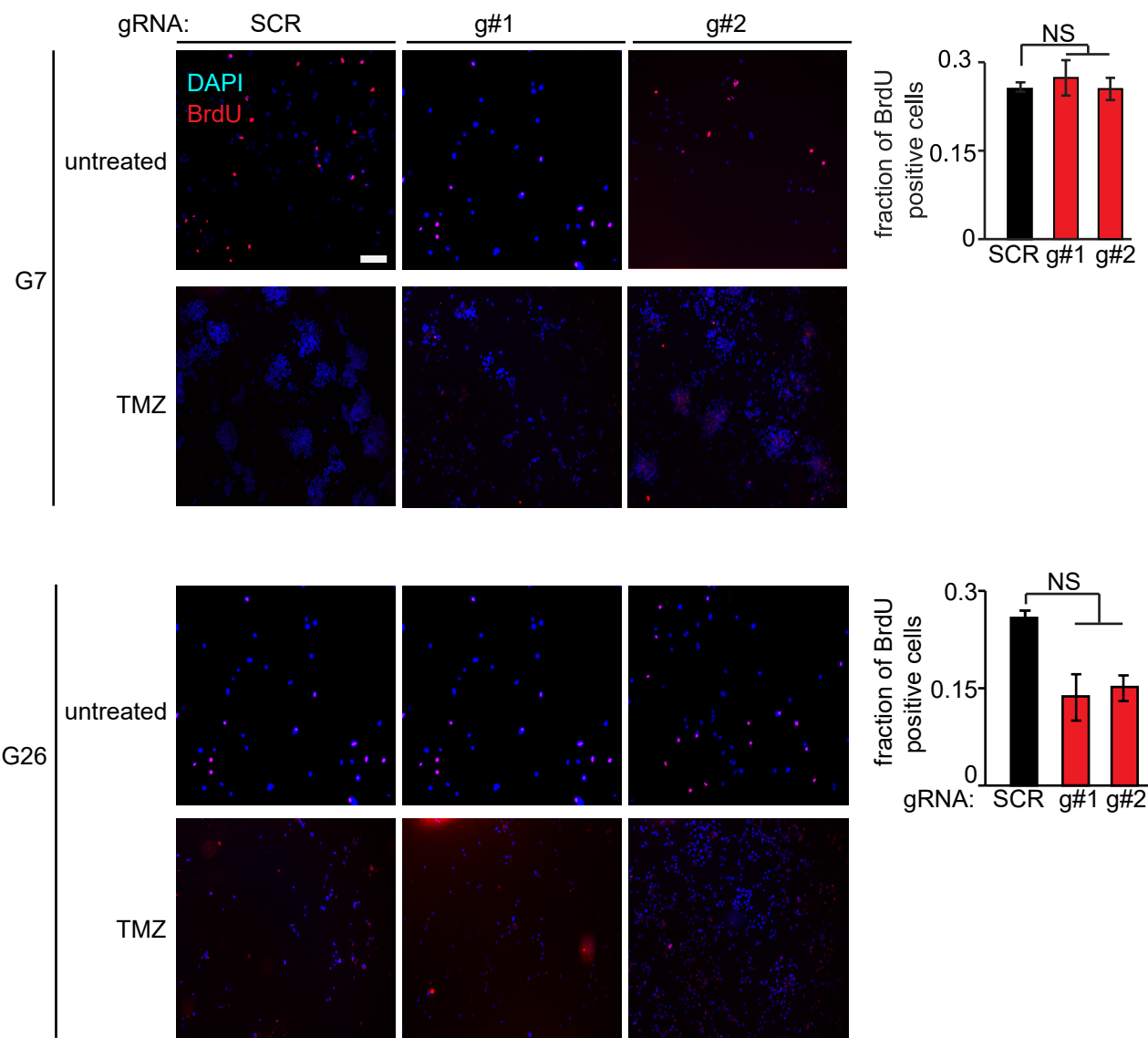

B

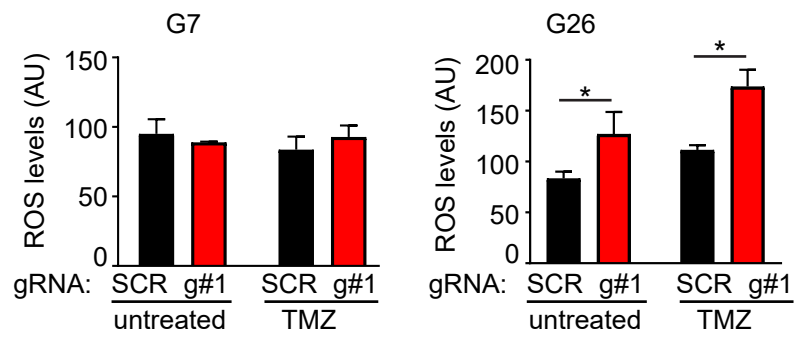

C

| sample | cg12434587 | cg12981137 | pred      | lower     | upper     | state |
|--------|------------|------------|-----------|-----------|-----------|-------|
| G7     | 4.3938844  | 5.255236   | 0.9999899 | 0.9976819 | 1         | M     |
| G26    | 0.6388169  | 6.107729   | 0.9999669 | 0.994969  | 0.9999998 | M     |

Supplement: Supplementary file 6 — SUP Figure 3 [file 41419_2019_1477_MOESM6_ESM.pdf]

A

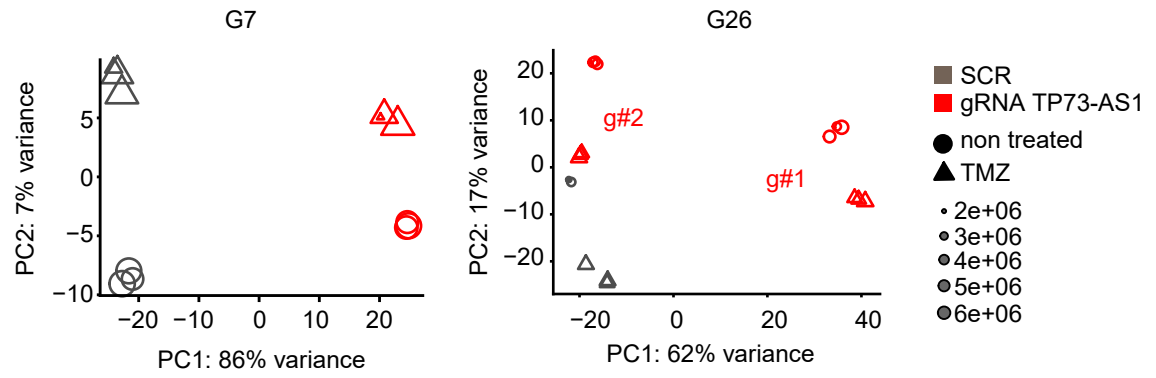

B

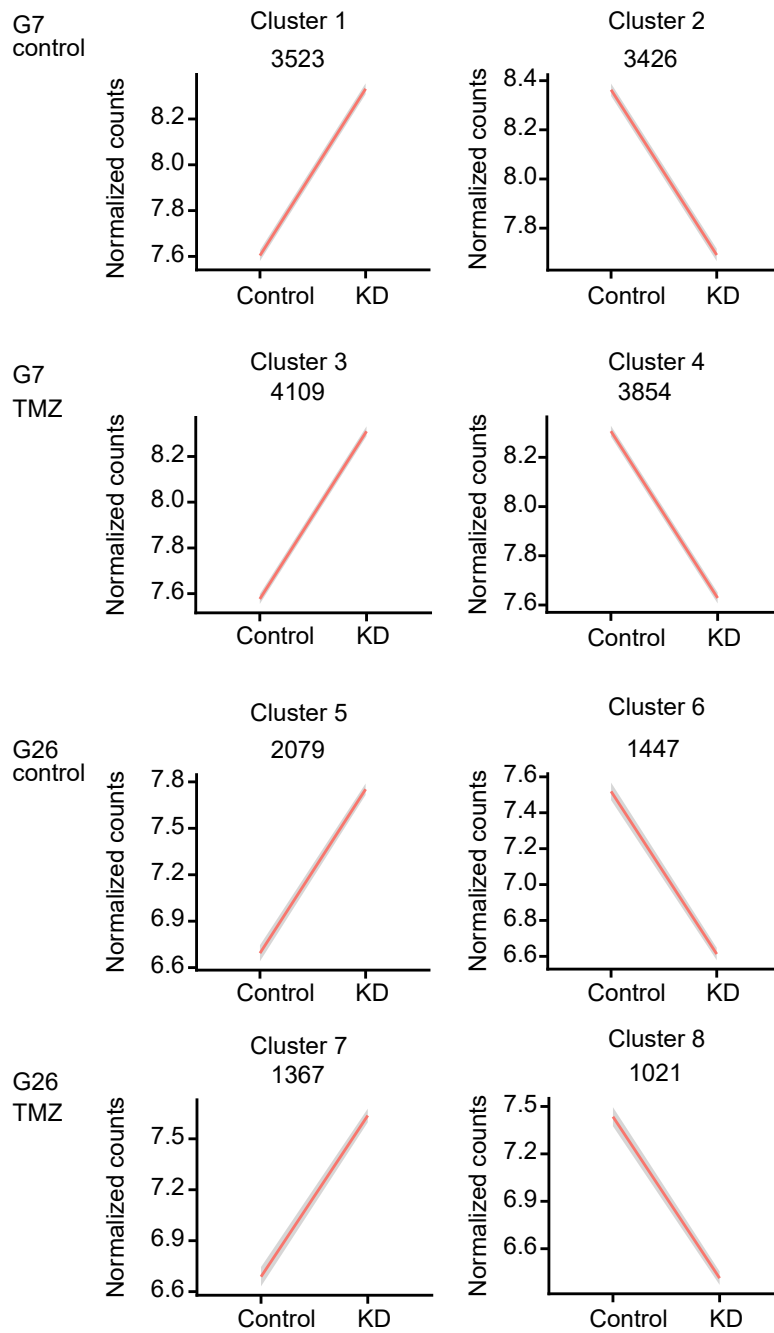

Supplement: Supplementary file 7 — SUP Figure 4 [file 41419_2019_1477_MOESM7_ESM.pdf]

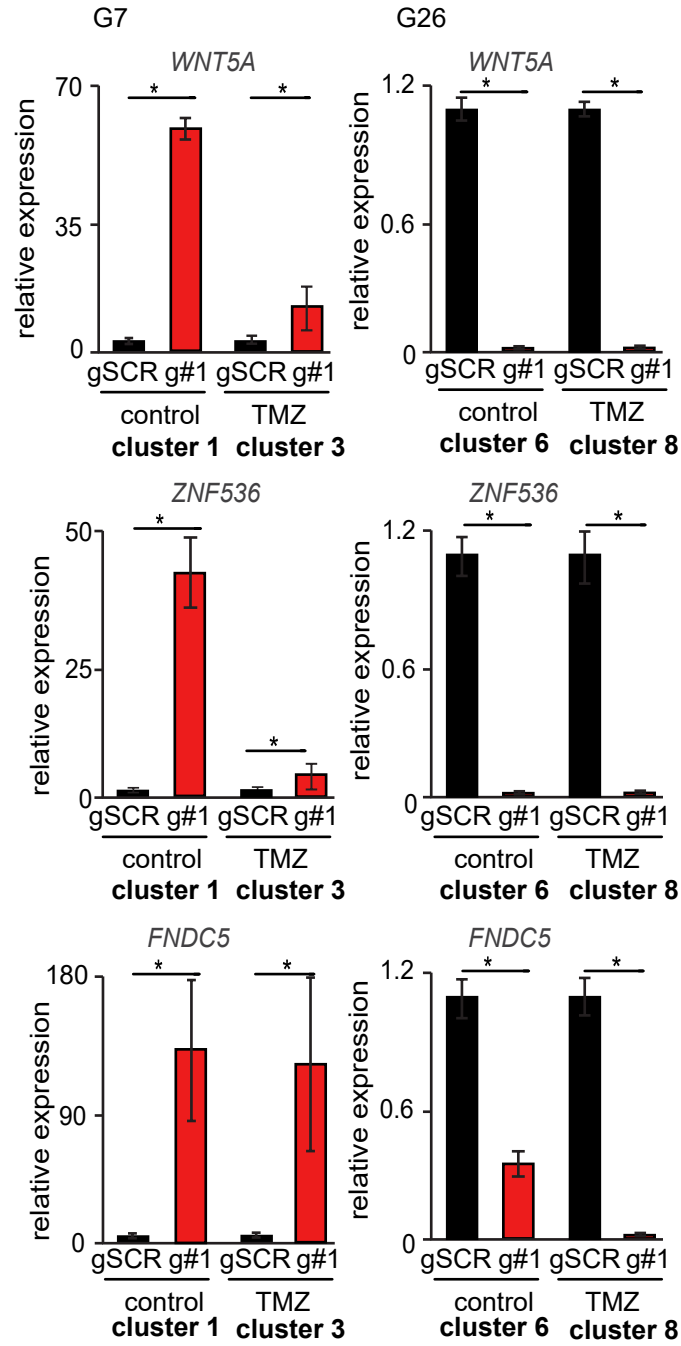

Supplement: Supplementary file 8 — SUP Figure 5 [file 41419_2019_1477_MOESM8_ESM.pdf]
